# Characterization of Lung Function Impairment in Adults with Bronchiectasis

**DOI:** 10.1371/journal.pone.0113373

**Published:** 2014-11-18

**Authors:** Wei-jie Guan, Yong-hua Gao, Gang Xu, Zhi-ya Lin, Yan Tang, Hui-min Li, Zhi-min Lin, Jin-ping Zheng, Rong-chang Chen, Nan-shan Zhong

**Affiliations:** 1 State Key Laboratory of Respiratory Disease, National Clinical Research Center for Respiratory Disease, First Affiliated Hospital of Guangzhou Medical University, Guangzhou, Guangdong, China; 2 Department of Respiratory and Critical Care Medicine, First Affiliated Hospital of Zhengzhou University, Zhengzhou, Henan, China; 3 Department of Geriatrics, Guangzhou First People’s Hospital, Guangzhou, Guangdong, China; University of Texas Health Science Center at Tyler, United States of America

## Abstract

**Background:**

Characteristics of lung function impairment in bronchiectasis is not fully understood.

**Objectives:**

To determine the factors associated with lung function impairment and to compare changes in spirometry during bronchiectasis exacerbation and convalescence (1 week following 14-day antibiotic therapy).

**Methods:**

We recruited 142 patients with steady-state bronchiectasis, of whom 44 with acute exacerbations in the follow-up were included in subgroup analyses. Baseline measurements consisted of chest high-resolution computed tomography (HRCT), sputum volume, purulence and bacteriology, spirometry and diffusing capacity. Spirometry, but not diffusing capacity, was examined during acute exacerbations and convalescence.

**Results:**

In the final multivariate models, having bronchiectasis symptoms for 10 years or greater (OR = 4.75, 95%CI: 1.46–15.43, *P* = 0.01), sputum culture positive for *Pseudomonas aeruginosa* (OR = 4.93, 95%CI: 1.52–15.94, *P*<0.01) and HRCT total score being 12 or greater (OR = 7.77, 95%CI: 3.21–18.79, *P*<0.01) were the major variables associated with FEV_1_ being 50%pred or less; and the only variable associated with reduced D_L_CO was 4 or more bronchiectatic lobes (OR = 5.91, 95%CI: 2.20–17.23, *P*<0.01). Overall differences in FVC and FEV_1_ during exacerbations and convalescence were significant (*P*<0.05), whereas changes in other spirometric parameters were less notable. This applied even when stratified by the magnitude of FEV_1_ and D_L_CO reduction at baseline.

**Conclusion:**

Significant lung function impairment should raise alert of chest HRCT abnormality and sputum culture positive for *Pseudomonas aeruginosa*, in patients with predominantly mild to moderate steady-state bronchiectasis. Acute exacerbations elicited reductions in FVC and FEV_1_. Changes of other spirometric parameters were less significant during exacerbations.

**Trial Registration:**

ClinicalTrials.gov NCT01761214

## Introduction

Bronchiectasis has been recognized as a debilitating disease with which patients frequently suffer from chronic cough, sputum production and recurrent infective exacerbations [1]. Persistent airway inflammation and mucus hypersecretion may predispose to mucus plugging and bronchial wall thickening and destruction, resulting in impaired lung function [2]. In bronchiectasis, airway obstruction and air trapping might be responsible for reduced diffusing capacity (D_L_CO) [3], which has been correlated with the degree of air trapping and reduction in FEV_1_ [2], suggesting that the combination of spirometry and diffusing capacity measurement may offer further information of functional impairment.

In patients with bronchiectasis, the lung function impairment might be heterogeneous and clinically associated with the number of bronchiectatic lobes, the magnitude of bronchial dilatation and dyshomogneity. There might be other factors that correlate with impaired lung function, for instance, the natural history of bronchiectasis. Ip *et al* [4] found that greater sputum volume, older age, diffuse disease and concomitant asthma were responsible for worse lung function. In another study, Roberts and colleagues [2] found that bronchial wall thickening and decreased attenuation on expiratory computed tomography (CT) signaled airflow obstruction. In addition, the effects of potentially pathogenic microorganisms (PPMs), in particular, *Pseudomonas aeruginosa* (*P. aeruginosa*) [5], on lung function decline have been recognized. However, it remains poorly elucidated which factors are associated with an increased likelihood of lung function impairment.

Furthermore, acute exacerbations are critical events in bronchiectasis leading to significantly worsened symptoms and impaired quality of life. It has been shown that patients with asthma or chronic obstructive pulmonary disease are characterized by pronounced lung function decline during exacerbations and recovery during convalescence. However, the underlying pathogenesis of bronchiectasis might result in a different magnitude of change in lung function.

We hypothesized that patients with worse clinical conditions – marked chest radiological abnormality and sputum isolation of PPMs – are associated with poorer lung function, and that patients with poorer spirometry when clinically stable were more likely to have dramatic reduction in spirometry during bronchiectasis exacerbations.

To test these hypotheses, we sought to investigate the magnitude of lung function impairment in clinically stable bronchiectasis and the association with the past history of bronchiectasis, radiology and sputum bacteriology, and to determine the changes in spirometry during acute exacerbations and convalescence.

## Methods

### Subjects

Subjects were recruited from the out-patient clinics of First Affiliated Hospital of Guangzhou Medical University. Diagnosis of bronchiectasis was based on chest high-resolution computed tomography with 1∼2 mm collimations and at 10 mm intervals within 12 months which was compatible with typical symptoms (chronic cough, sputum production or hemoptysis). Patients 18–75 years of age were eligible if clinically stable (cough frequency, sputum volume and purulence not significantly exceeding their normal daily variations, and lack of emerging onset of chest pain, dyspnea, hemoptysis or fever) for at least 4 weeks, confirmed by the attending study investigators. The clinical stability was verified by careful review of the medical charts since the date of out-patient clinic physician’s referral and interview with the patients. Exclusion criteria consisted of malignancy; use of oral/systemic antibiotics/corticosteroids for the treatment of bronchiectasis within 4 weeks; limited understanding.

The study protocol was approved by Ethic Committee of The First Affiliated Hospital of Guangzhou Medical University and subjects gave written informed consent.

### Study design

Study 1 was a cross-sectional study, conducted between September 2012 and October 2013, investigating the magnitude of lung function impairment and the relationships with radiology and sputum bacteriology, as well as the risk factors of lung function impairment. Patients underwent spirometry, measurement of diffusing capacity and sputum induction.

Study 2 was a prospective follow-up study, conducted between November 2012 and October 2013, in which the changes in spirometry during steady-state, acute exacerbation and convalescence were compared. Convalescence was defined as 1 week after cessation of 14-day antibiotic therapy. Patients were informed to contact the investigators in cases of worsened respiratory symptoms that warranted prompt hospital visits for assessments. Acute exacerbation denoted the significant changes (beyond normal daily variations) in three or more of the following symptoms/signs for at least 2 days: cough frequency, 24-hour sputum volume, sputum purulence and (or) emerging fever, dyspnea or chest pain. Antibiotics were prescribed according to British Thoracic Guideline for Bronchiectasis [6] by integrating previous results of sputum bacteriology. Patients were requested to undertake reassessments at convalescence stage (at 1 week following 14-day antibiotic treatment), regardless of treatment outcomes. Measurements of exacerbation and convalescence visits comprised spirometry, sputum bacteriology, serum and sputum biomarkers, and quality of life (details other than spirometry will be reported elsewhere). If more than one exacerbation for an individual patient was reported, only the first one was included in our analysis.

Despite that the local Ethics Committee’s approval for submitting the English version of our study protocol to clinical trial registry took place after patient recruitment, we confirmed that the two versions shared identical contents and that we have consistently adhered to the approved Chinese protocol ever since. The authors also confirm that all ongoing and related trials for this drug/intervention are registered.

The protocol for this trial and supporting TREND checklist are available as supporting information; see Checklist S1 and Protocol S1.

### Spirometry

Spirometry was conducted by using spirometers (QUARK PFT, COSMED Inc., Milan, Italy). The quality control met guidelines for standardization [7]. Between-maneuver variation was less than 5% or 200 ml in FVC and FEV_1_, with the maximal values being reported. Maximal mid-expiratory flow (MMEF), mid-expiratory flow at 50% (MEF_50%_) and 25% lung volume (MEF_25%_) were chosen from the best maneuver. Predicted values were selected using the reference model by Zheng et al [8]. Salbutamol was withdrawn for at least 6 hours and salmeterol or formoterol 24 hours prior to spirometry.

### Assessment of chest HRCT scores

Chest HRCT at collimation of 2 mm or less within 12 months was captured. Bronchiectasis was diagnosed if the internal diameter of bronchi was greater than accompanying pulmonary artery. Miscellaneous signs of bronchiectasis included the lack of normal bronchial tapering along travel on sequential slices and (or) visible bronchi within 10 mm to the pleura. The HRCT score was assessed on a lobar basis, with lingular lobe being regarded as a separate lobe. For an individual lung lobe, the radiological severity of bronchiectasis were scaled by using modified Reiff score [9]. The maximal total score was 18 for a total of six lobes. Miscellaneous characteristics of HRCT, including the predominant bronchiectatic lobes, dyshomogneity, atelectasis and infiltration, were also determined.

### Measurement of diffusing capacity

Diffusing capacity was measured with gas analyzers (QUARK PFT, COSMED Inc., Milan, Italy) by using single-breath carbon monoxide washout technique. Ingestion of food, exercising, smoking and alcohol drinking was withheld for 24 hours. Calibration using standardized gases was performed each day, prior to measurement. Subjects wearing nose clip, in seated position, were instructed to breathe tidally via the mouthpiece. This was followed by thorough exhalation to residual volume followed by rapid (<2.5 seconds) and complete (≥90% vital capacity) inhalation to total lung capacity. Following 10-second breath-holding maneuver, subjects exhaled at moderate airflow to residual volume. Artifacts, including Valsava or Muller’s maneuver or hesitation during rapid inhalation, should be avoided. The interval between repetitive measurements was 4 minutes, and measurements were deemed acceptable with a coefficient of variation for D_L_CO of less than 10% or the absolute variation of less than 3 ml/(min*mmHg). Both D_L_CO and D_L_CO/V_A_ were reported based on the mean of two maneuvers. Reduced diffusing capacity denoted D_L_CO of 80% predicted or less.

### 24-hour sputum volume assessment

At the initial hospital visit, patients were requested to collect 24-hour sputum in a clear plastic pot, for 3 consecutive days, when clinically stable. Sputum volume was recorded to the nearest 5 ml. The mean of three readings was reported and artificially categorized into 10 ml or less, 11 to 29 ml, and 30 ml or greater.

### Sputum culture and quantitative assessment of bacterial load

Fresh sputum sample was collected during hospital visits between 9:00am and 11:00 am. Subjects were instructed to fully empty their mouth and remove oral remnants followed by chest physiotherapy by a designated research nurse for 10 minutes. This was followed by forced expectoration to collect sputum sample into a 50 ml clear sterile plastic pot.

Methods for assessment of sputum bacteriology have been introduced previously [1]. Briefly, blood agar and chocolate agar plates (Biomeurix Co. Ltd., France) were adopted for culture. Fresh sputum was homogenized with SPUTASOL (Oxoid SR089A) and serially diluted with natural saline (10^−4^, 10^−5^ and 10^−6^). This was followed by addition of 10 µl respective diluent to agar plates with micropipette tube and the inoculation using 10 µl standardized rings. Plates were positioned in thermostatic box containing 5% carbon dioxide at 37°C for overnight incubation.

PPMs were categorized as *P. aeruginosa*, *Hemophilus spp.* and miscellaneous PPMs (*Stenotrophomonas maltophilia, Staphylococcus aureus, Escherichia colitis, Sphingomonas paucimobilis, Klebsiella spp., Alcaligenes faecalis subsp faecalis, Psedumonas pseudoalcaligenes and Serratia marcescens*). Non-PPMs (commensals) included *Neisseria,* α*-Streptococcus hemolyticus, Bacilli diphtheria* and coagulase-negative staphylococcus. Prolonged culture was done for negative plates. Bacterial load, measured as colony forming unit per milliliter (cfu/ml), was reported for PPMs only. Only the plates with 30 to 300 cfu were counted for bacterial load.

In our study, bacterial colonization (referred to as infection specifically for *P. aeruginosa*) was defined as sputum culture positive of an identical PPM for at least 2 occasions within 1 year, at least 3 months apart. Bacterial isolation denoted sputum culture positive of PPMs at baseline.

### Statistical analysis

Statistical analyses were performed using SPSS 16.0 package (SPSS Inc., Chicago, IL., USA). Numerical data were presented as mean ± standard deviation (SD) or median (interquartile range, IQR) as appropriate. Between-group differences were compared using independent t-test or Mann-Whitney test as indicated; one-way analysis of variance (ANOVA) or Kruskal-Wallis test were applied for comparisons of more than two groups as appropriate. The difference in spirometry at exacerbation and convalescence visit as compared with steady-state was compared using paired t-test or Mann-Whitney test as appropriate. *Post hoc* analysis was done using SNK test. The categorical data were expressed as the absolute (percentage) and compared using chi-square test. Logistic regression analysis was conducted to examine the risk factors of FEV_1_<50% predicted and D_L_CO<80% predicted, respectively. For univariate analyses, the odds ratios (OR) for FEV_1_<50% predicted and D_L_CO<80% predicted were estimated with 95% confidence intervals (95%CIs). Determinants with *P* value of 0.20 or less in the univariate models were entered into multivariate analysis and discarded using backward selection technique. Following adjustment, the variables with P value of 0.05 or less were deemed as the final risk factors. All comparisons were two-sided, and *P*<0.05 was taken as statistically significant.

## Results

### Subject enrollment

**Figure 1:**
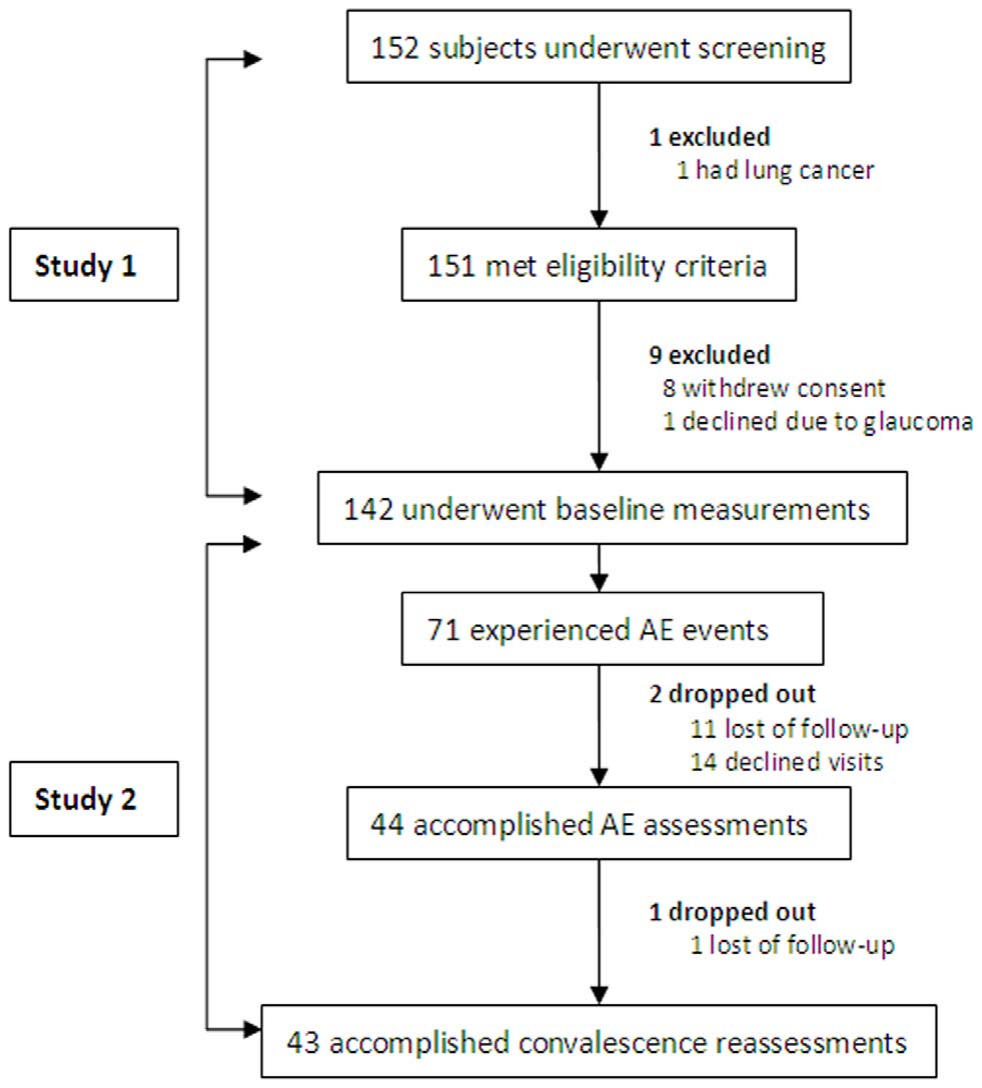
Subject enrollment flowchart. Of 151 patients underwent screening, 151 met eligibility criteria and 142 were included for analysis of study 1. Of these patients, 71 reported AEs, and finally 43 patients accomplished convalescence measurements. The data derived from 142 patients who underwent baseline measurements were analyzed for study 1 and 44 patients who accomplished assessments of acute exacerbations for study 2, respectively.

Subject enrollment is detailed in Figure 1. The data derived from 142 patients who underwent baseline measurements were analyzed for study 1 and 44 patients who accomplished assessments of acute exacerbations for study 2, respectively.

### Baseline levels

As shown in Table 1, both cohorts (all patients for baseline assessments and patients with reported-exacerbations) had similar profiles of anthropometric characteristics, past history, disease severity, medications ever used within 6 months and the associated findings. The spectra of sputum bacteriology, predominated by commensals and *P. aeruginosa*, were similar. Diffusing capacity was not performed at baseline in 13 patients due to poor compliance (n = 7) and an FVC of 1.0 liter or less (n = 6). The most common medication used within 6 months was mucolytics, followed by xanthenes, macrolides and inhaled corticosteroids.

**Table 1:** Baseline characteristics of bronchiectasis patients.

Parameter	Study 1 (n = 142)	Study 2 (n = 44)
**Anthropometry**		
Age (years)	44.7±13.9	42.6±13.5
Females (No., %)	88 (62.0)	27 (61.4)
Height (cm)	160.0 (10.2)	161.6±7.3
Weight (kg)	51.5 (10.5)	51.7±9.0
BMI (kg/m^2^)	19.9 (4.0)	19.8±3.2
Never-smokers (No., %)	122 (85.9)	36 (81.8)
**Past history**		
Duration of bronchiectasis symptoms (yrs)	10.0 (16.0)	10.0 (14.0)
Duration of diagnosis (yrs)	3.0 (9.0)	5.0 (9.0)
No. of acute exacerbations within 2 yrs		
0–1 episode (No., %)	33 (23.2)	8 (18.2%)
2–3 episodes (No., %)	43 (30.3)	16 (36.5%)
4 or more episodes (No. %)	66 (46.5)	20 (45.5%)
**HRCT findings**		
No. of bronchiectatic lobes	4.0 (2.0)	4.0 (3.0)
HRCT total score	7.0 (5.0)	7.0 (7.0)
**Baseline sputum bacteriology**		
* Pseudomonas aeruginosa* (No., %)	42 (29.6)	16 (36.4)
* Hemophilus influenzae* (No., %)	11 (7.7)	2 (4.5)
* Hemophilus parainfluenzae* (No., %)	16 (11.3)	4 (9.1)
Other PPMs (No., %)*	14 (9.9)	5 (11.4)
Commensals (No., %)	59 (41.5)	17 (38.6)
**Medications ever taken within 6 months**		
Xanthenes (No., %)	84 (59.2)	27 (61.4)
Inhaled corticosteroids (No., %)	32 (22.5)	10 (22.7)
Mucolytics (No., %)	106 (74.6)	39 (88.6)
Macrolides (No., %)	58 (40.8)	17 (38.6)
**Associated findings** **		
Post-infectious (No., %)	38 (26.8)	11 (25.0)
Immunodeficiency (No., %)	17 (12.0)	6 (13.6)
Asthma (No., %)	7 (4.9)	2 (4.6)
Gastroesophageal reflux (No., %)	6 (4.2)	3 (6.8)
Miscellaneous known findings (No., %)	22 (15.5)	8 (18.2)
Idiopathic (No., %)	64 (45.1)	17 (38.6)

Numerical data were presented as mean ± standard deviation (SD) or median (interquartile range, IQR) as appropriate.

NA: not applicable. AE: acute exacerbation.

* Other pathogenic bacteria included *Stenotrophomonas maltophilia* (n = 2, 1.4%), *Staphylococcus aureus* (n = 4, 2.8%), *Escherichia colitis* (n = 1, 0.7%), *Sphingomonas paucimobilis* (n = 1, 0.7%), *Klebsiella ozaenae* (n = 1, 1.4%), *Klebsiella pneumonae* (n = 2, 1.4%), *Alcaligenes faecalis subsp faecalis* (n = 1, 0.7%), *Psedumonas pseudoalcaligenes* (n = 1, 0.7%) and *Serratia marcescens* (n = 1, 0.7%).

The associated findings of bronchiectasis were determined after meticulous testing recommended by British Thoracic Society guidelines and group discussion (W.G., Y.G. and G.X.). Further details will be published elsewhere.

** Dual associated findings existed in some individuals, and therefore the percentage added up to 100% or greater. Miscellaneous findings included COPD, rheumatoid arthritis, lung malformation, lung sequestration, yellow nail syndrome, Young’s syndrome and eosinophilic bronchiolitis.

Because of the scarcity of cases with cystic fibrosis ever reported in Asian countries, we did not conduct routine screening using sweat test or genetic workup. Consequently, our cohort phenotypically represented a mixture of non-cystic fibrosis bronchiectasis.

### Association between lung function impairment and clinical indices

Associations between lung function impairment and clinical indices are presented in Table 2. *P. aeruginosa* isolated from sputum, PPMs colonization (including *P. aeruginosa* infection), higher HRCT total scores, higher 24-hour sputum volume, more bronchiectatic lobes, bilateral bronchiectasis, and the presence of cystic bronchiectasis, dyshomogneity and pulmonary infiltrations were associated with poorer FEV_1_ (all *P*<0.05). Sputum purulence was not correlated with FEV_1_, except for mucoid sputum (*P*<0.01).

**Table 2:** Lung function characteristics based on clinical characteristics.

Parameter	Spirometry				Diffusing capacity*		
	FEV_1_pred≥80% (n = 54)	80%<FEV_1_pred≤50% (n = 59)	FEV_1_<50%pred (n = 29)	P	DLCO≥80%pred (n = 98)	DLCO<80%pred (n = 31)	P
**Sputum bacteriology**							
* Pseudomonas aeruginosa*	**12 (22.2)**	**15 (25.4)**	**15 (51.7)**	**0.01**	**21 (21.4)**	**16 (51.6)**	**<0.01**
* Hemophilus spp*	8 (14.8)	13 (22.0)	6 (20.7)	0.60	20 (20.4)	4 (12.9)	0.35
Miscellaneous PPMs	6 (11.1)	6 (10.2)	2 (6.9)	0.82	10 (10.2)	4 (12.9)	0.67
Commensals	**28 (51.9)**	**25 (42.4)**	**6 (20.7)**	**0.02**	**46 (46.9)**	**7 (22.6)**	**0.02**
**Bacterial colonization** **							
Yes	**7 (13.0)**	**16 (27.1)**	**13 (44.8)**	**<0.01**	**22 (22.4)**	**10 (32.3)**	**0.27**
Nil	**47 (87.0)**	**43 (72.9)**	**16 (55.2)**	**<0.01**	**76 (77.6)**	**21 (67.7)**	**0.27**
**HRCT score**							
1∼6	**40 (74.1)**	**25 (42.4)**	**3 (10.3)**	**<0.01**	54 (55.1)	11 (35.5)	0.06
7∼13	13 (24.1)	22 (37.3)	14 (48.3)	0.07	40 (40.8)	12 (38.7)	0.83
14∼18	**1 (1.9)**	**2 (3.4)**	**12 (41.4)**	**<0.01**	**4 (4.1)**	**8 (25.8)**	**<0.01**
**HRCT findings**							
≤ 3 bronchiectatic lobes	**41 (75.9)**	**20 (33.9)**	**4 (13.8)**	**<0.01**	**56 (57.1)**	**5 (16.1)**	**<0.01**
>4 bronchiectatic lobes	**13 (24.1)**	**39 (66.1)**	**25 (86.2)**	**<0.01**	**42 (42.9)**	**26 (83.9)**	**<0.01**
Predominantly upper lobe bronchiectasis	1 (1.9)	0 (0.0)	0 (0.0)	0.27	1 (1.0)	0 (0.0)	1.00
Predominantly middle/lower lobe bronchiectasis	33 (61.1)	46 (78.0)	22 (75.9)	0.12	68 (69.4)	25 (80.6)	0.22
Unilateral bronchiectasis	**15 (27.8)**	**9 (15.3)**	**0 (0.0)**	**<0.01**	21 (21.4)	2 (6.5)	0.10
Bilateral bronchiectasis	**39 (72.2)**	**50 (84.7)**	**29 (100.0)**	**<0.01**	77 (78.6)	29 (93.5)	0.10
Nil cystic bronchiectasis	**33 (61.1)**	**27 (45.8)**	**3 (10.3)**	**<0.01**	48 (49.0)	11 (35.5)	0.22
Cystic bronchiectasis	**21 (38.9)**	**32 (54.2)**	**26 (89.7)**	**<0.01**	50 (51.0)	20 (64.5)	0.13
Nil dyshomogneity	**31 (57.4)**	**18 (30.4)**	**2 (6.9)**	**<0.01**	**43 (43.9)**	**6 (19.4)**	**0.02**
Dyshomogneity	**23 (42.3)**	**41 (69.5)**	**27 (93.1)**	**<0.01**	**55 (56.1)**	**25 (80.6)**	**0.01**
Nil atelectasis	44 (81.5)	41 (69.5)	19 (65.5)	0.20	69 (70.4)	23 (74.2)	0.82
Atelactasis	10 (18.5)	18 (30.5)	10 (34.5)	0.20	29 (29.6)	8 (25.8)	0.68
Nil infiltration	**10 (18.5)**	**4 (6.8)**	**0 (0.0)**	**0.02**	13 (13.3)	1 (3.2)	0.74
Infiltration	**44 (81.5)**	**55 (93.2)**	**29 (100.0)**	**0.02**	85 (86.7)	30 (96.8)	0.22
**24-hour sputum volume (ml)**							
<10	**31 (57.4)**	**22 (37.3)**	**4 (13.8)**	**<0.01**	**38 (38.8)**	**6 (19.4)**	**0.02**
11∼29	17 (31.5)	23 (39.0)	14 (48.3)	0.32	43 (43.9)	9 (29.0)	0.14
>30	**6 (11.1)**	**14 (23.7)**	**11 (37.9)**	**0.02**	**17 (17.3)**	**16 (51.6)**	**<0.01**
**Sputum characteristics**							
Mucoid	**25 (46.3)**	**11 (18.6)**	**4 (13.8)**	**<0.01**	**33 (33.7)**	**4 (12.9)**	**0.03**
Mucopurulent	23 (42.3)	34 (57.6)	16 (55.2)	0.25	49 (50.0)	16 (51.6)	0.88
Purulent	6 (11.1)	14 (23.7)	9 (31.0)	0.07	**16 (16.3)**	**11 (35.5)**	**<0.01**
**Exacerbations within 2 yrs**							
0–1	**17 (31.5)**	**14 (23.7)**	**2 (6.9)**	**0.04**	**27 (27.6)**	**2 (6.5)**	**0.03**
2–3	18 (33.3)	17 (28.8)	8 (27.6)	0.82	32 (32.7)	6 (19.5)	0.13
≥4	**19 (35.2)**	**28 (47.5)**	**19 (65.5)**	**0.03**	**39 (39.8)**	**23 (59.0)**	**<0.01**

All data are expressed as No. (%) unless otherwise stated.

* Subjects with missing D_L_CO values were not included in the final statistical analyses.

P values were derived from the chi-square tests between the two groups (for diffusing capacity) or among the three groups (for spirometry).

** In our study, bacterial colonization (referred to as infection specifically for P. *aeruginosa*) was defined as sputum culture positive of an identical PPM for at least 2 occasions within 1 year, at least 3 months apart. Bacterial isolation denoted sputum culture positive of PPMs at baseline.

Reduced D_L_CO was associated with *P. aeruginosa* isolated from sputum, higher 24-hour sputum volume and purulence score, more frequent exacerbations, more bronchiectatic lobes and the presence of dyshomogneity (all *P*<0.05). The HRCT total score, with exception of the 14-to-18-point subgroup, was a poor predictor of reduced D_L_CO.

The lung function parameters stratified by clinical indices can also be seen in Table S1 in File S1.

### Factors associated with FEV_1_<50%pred and D_L_CO<80%pred

Univariate analysis indicated that having physician-diagnosed bronchiectasis for 3 years or greater, 24-hour sputum being 30 ml or greater, higher HRCT total score, more bronchiectatic lobes, presence of cystic bronchiectasis and dyshomogneity, sputum culture positive for *P. aeruginosa* were the factors associated with FEV_1_<50%pred (all *P*<0.01). When observed with reduced diffusing capacity, the associated factors consisted of having bronchiectasis symptoms for 10 years or greater, 24-hour sputum being 10–30 ml, four or more acute exacerbations within 2 years, purulent sputum, HRCT total score being 13 or greater, four or more bronchiectatic lobes, presence of dyshomogneity and sputum culture positive for *P. aeruginosa* (all *P*<0.05). (see Table S3 in File S1).

Multivariate analyses (Table 3) revealed that having bronchiectasis symptoms for 10 years or greater (OR = 4.75, 95%CI: 1.46–15.43, *P* = 0.01), sputum culture positive for *P. aeruginosa* (OR = 4.93, 95%CI: 1.52–15.94, *P*<0.01) and HRCT total score being 13 or greater (OR = 7.77, 95%CI: 3.21–18.79, *P*<0.01) were associated with FEV_1_ 50%pred or less. The only factor associated with reduced D_L_CO was 4 or more bronchiectatic lobes (OR = 5.91, 95%CI: 2.20–17.23, *P*<0.01).

**Table 3:** Multivariate model for the factors associated with FEV_1_<50%pred and D_L_CO<80%pred.

Parameter	FEV_1_<50%pred			D_L_CO<80%pred		
	OR	95%CI	P	OR	95%CI	P
**No. of bronchiectatic lobes**	-	-	-	5.91	2.20–17.23	<0.01
**Duration of symptoms**	4.75	1.46–15.43	0.01	-	-	-
***Pseudomonas aeruginosa*** ** isolated from sputum**	4.93	1.52–15.94	<0.01	-	-	-
**HRCT total score**	7.77	3.21–18.79	<0.01	-	-	-

### Spirometry during steady-state, acute exacerbation and convalescence of bronchiectasis when stratified by FEV_1_ and D_L_CO reduction

See online supplement for the category of antibiotics (also see Table S2 in File S1). Overall, there was significant reduction in FVC and FEV_1_ at exacerbations and recovery during convalescence (*P*<0.05). (Table 4) Subgroup analyses showed similar findings in patients with different magnitudes of FEV_1_ and D_L_CO impairment. Changes in miscellaneous spirometric parameters were less notable. Patients isolated with *P. aeruginosa* also demonstrated similar trends of changes in spirometric parameters (see Figure S1 in File S1). Although there was a trend towards a reduction during acute exacerbations and recovery during convalescence, the mean changes in spirometry were 5% or less in most patients, compared with baseline levels.

**Table 4:** Comparison on changes in spirometry in acute exacerbation and convalescence of bronchiectasis stratified by the magnitude of FEV_1_ and D_L_CO reduction.

	Parameter	Baseline	Acute exacerbation			Convalescence		
			Mean ± SD	P	Change from baseline (Mean, 95%CI)	Mean ± SD	P’	Change from exacerbation (Mean, 95%CI)
**All subjects (n = 44)**	**FVC pred%**	**78.69±23.16**	**75.97±24.09**	**0.02**	**−2.72 (−5.20, −0.23)**	**79.80±23.41**	**0.01**	**0.94 (0.51, 5.93)**
	**FEV_1_ pred%**	**66.59±23.65**	**64.05±24.56**	**0.01**	**−2.54 (−4.74, −0.33)**	**68.04±23.70**	**0.03**	**3.62 (0.43, 6.81)**
	**FEV_1_/FVC**	70.18±12.86	69.80±13.91	0.33	−0.39 (−2.13, 1.35)	70.71±12.12	0.32	1.16 (−1.17, 3.49)
	**MMEF pred%**	**42.14 (37.29)**	**44.56±25.86**	**0.05**	**−3.18 (−6.91, 0.55)**	47.75±26.23	0.12	2.74 (0.71, 6.18)
	**MEF_50%_ pred%**	40.13±24.15	39.50±23.93	0.34	−0.64 (−3.79, 2.51)	40.83±23.05	0.53	0.99 (−2.13, 4.11)
	**MEF_25%_ pred%**	38.63±20.80	33.45 (24.12)	0.27	1.77 (−3.94, 7.47)	35.89±19.41	0.32	−2.96 (−8.95, 3.03)
**FEV_1_≥80%pred (n = 15)**	**FVC pred%**	97.64±12.85	96.33±17.96	0.29	−1.31 (−6.30, 3.69)	93.80 (21.61)	0.33	0.78 (−2.96, 4.53)
	**FEV_1_ pred%**	**87.69 (8.42)**	**87.36±15.86**	**0.05**	**−3.72 (−8.29, 0.84)**	89.86±14.09	0.10	2.50 (−1.48, 6.47)
	**FEV_1_/FVC**	**78.42±7.49**	**76.21±8.83**	**0.05**	**−2.21 (−4.91, 0.49)**	77.63±7.20	0.16	1.41 (−1.48, 4.30)
	**MMEF pred%**	**72.14±24.05**	**65.41±25.20**	**0.02**	**−6.73 (−13.32, −0.14)**	70.80±22.90	0.08	5.39 (−2.48, 13.26)
	**MEF_50%_ pred%**	63.45±20.13	58.97±23.05	0.12	−4.48 (−12.47, 3.51)	61.04±16.95	0.31	2.07 (−6.82, 10.96)
	**MEF_25%_ pred%**	55.86±19.41	51.62 (31.95)	0.36	2.98 (−14.41, 20.37)	53.36±16.62	0.26	−5.48 (−23.20, 12.23)
**80%pred <FEV_1_≤50% pred (n = 17)**	**FVC pred%**	**81.57±14.87**	**76.85±14.77**	**0.01**	**−4.87 (−9.03, 0.70)**	**80.74±16.06**	**0.02**	**3.89 (0.33, 7.46)**
	**FEV_1_ pred%**	67.02±9.06	64.86±12.87	0.09	−2.44 (−6.13, 1.25)	66.46±12.54	0.13	1.60 (1.35, 4.54)
	**FEV_1_/FVC**	70.16±11.25	71.32±11.68	0.17	1.12 (−1.28, 3.52)	69.86±11.35	0.05	−1.46 (−3.14, 0.23)
	**MMEF pred%**	40.24 (24.40)	44.05±14.32	0.17	−3.31 (−10.50, 3.89)	42.90±15.05	0.20	−1.15 (−4.03, 1.74)
	**MEF_50%_ pred%**	37.27±15.15	38.56±14.25	0.14	1.24 (−1.11, 3.58)	37.87±15.76	0.27	−0.69 (−3.05, 1.67)
	**MEF_25%_ pred%**	34.23±12.56	35.54±8.90	0.31	0.85 (−2.62, 4.31)	**33.13±11.08**	**0.02**	**3.41 (−6.50, 0.33)**
**FEV_1_<50%pred (n = 12)**	**FVC pred%**	53.01±19.18	51.57±18.25	0.25	−1.44 (−5.93, 3.05)	56.99±20.23	0.08	5.42 (−2.54, 13.38)
	**FEV_1_ pred%**	36.39±11.41	35.20±12.78	0.26	−1.19 (−5.20, 2.82)	42.74±17.03	0.07	7.54 (−2.79, 17.87)
	**FEV_1_/FVC**	59.25±13.70	59.01±17.09	0.46	−0.25 (−5.06, 4.57)	63.13±13.73	0.13	4.12 (−3.47, 11.71)
	**MMEF pred%**	19.29±10.48	14.12 (12.95)	0.30	1.45 (−4.32, 7.21)	18.50 (28.72)	0.11	4.28 (−3.01, 11.56)
	**MEF_50%_ pred%**	16.03±8.38	12.14 (8.56)	0.30	1.51 (−4.63, 7.65)	13.17 (7.34)	0.09	1.74 (−0.88, 4.35)
	**MEF_25%_ pred%**	16.46±6.99	18.01±12.84	0.23	1.55 (−3.01, 6.10)	14.60 (11.11)	0.23	0.74 (−1.39, 2.87)
**DLCO≥80%pred (n = 24)**	**FVC pred%**	**92.47±13.39**	**88.06±19.07**	**0.01**	**−4.54 (−8.44, −0.64)**	89.99±16.49	0.13	1.93 (−1.59, 5.44)
	**FEV_1_ pred%**	**79.36±18.12**	**75.59±22.25**	**0.02**	**−3.84 (−7.48, −0.20)**	76.88±20.75	0.14	1.30 (−1.18, 3.77)
	**FEV_1_/FVC**	71.66±12.39	71.02±11.62	0.30	−0.52 (−2.57, 1.54)	70.86±12.19	0.43	−0.16 (−1.96, 1.64)
	**MMEF pred%**	**55.94±27.95**	**52.00±26.88**	**0.03**	**−3.77 (−7.85, 0.30)**	53.60±27.31	0.22	1.60 (−2.56, 5.77)
	**MEF_50%_ pred%**	48.74±24.99	47.42±24.60	0.31	−1.15 (−5.99, 3.70)	47.41±22.99	0.50	0.01 (−5.29, 5.28)
	**MEF_25%_ pred%**	42.78±22.01	37.04 (24.30)	0.26	3.17 (−7.07, 13.42)	40.22±19.76	0.13	−6.27 (−17.42, 4.89)
**DLCO<80%pred (n = 14)**	**FVC pred%**	66.08±25.36	64.92±25.62	0.25	−1.16 (−4.81, 2.49)	**68.35±24.47**	**0.04**	**3.43 (−0.36, 7.22)**
	**FEV_1_ pred%**	56.14±22.62	55.34±22.39	0.27	−0.80 (−3.55, 1.96)	**59.80±22.96**	**0.02**	**4.45 (0.08, 8.83)**
	**FEV_1_/FVC**	71.16±13.75	71.76±16.50	0.36	0.61 (−3.04, 4.25)	73.29±12.27	0.21	1.53 (−2.49, 5.55)
	**MMEF pred%**	40.77±23.74	41.91±23.77	0.36	1.14 (−5.49, 7.76)	44.38±23.38	0.22	2.47 (−4.15, 9.09)
	**MEF_50%_ pred%**	35.53±21.92	36.13±22.36	0.42	0.60 (−5.57, 6.77)	39.00±21.50	0.12	2.87 (−2.25, 8.00)
	**MEF_25%_ pred%**	32.32±18.31	34.13±19.38	0.25	1.82 (−3.83, 7.46)	34.73±17.60	0.41	0.60 (−5.13, 6.33)

Data in bold indicated statistical significance.

P’: P value of convalescence level compared with that of acute exacerbation. Subjects with missing D_L_CO values were not included in the final analyses.

P values were derived from paired t-test or Mann-Whitney test as appropriate.

## Discussion

Our data showed that lung function impairment was associated with aberrant chest radiologic findings and sputum bacteriology (esp. *P. aeruginosa*) in clinically stable bronchiectasis. Significant clinical impairment should raise the alert of poorer FEV_1_ and D_L_CO. We further showed that FVC and FEV_1_ were significantly reduced during acute exacerbations and recovered during convalescence, even when stratified by the magnitude of FEV_1_ and D_L_CO impairment. Other spirometric parameters vary less significantly during bronchiectasis exacerbations.

Sputum bacteriology was, in part, associated with poorer spirometry and (or) diffusing capacity. Infection with *P. aeruginosa*, but not colonization with miscellaneous bacteria, has been linked to poorer lung function [5]. Compared with nil colonization or intermittent isolation, the chronic *P. aeruginosa* infection has been shown to signal poorer FEV_1_, FEV_1_/FVC, D_L_CO and a higher level of residual volume [10], indicating the presence of airway obstruction and dyshomogneity. Chronic colonization by *P. aeruginosa* has also been associated with more rapid lung function decline [11]. In a more recent literature by Rogers *et al* [12], bronchiectasis patients with *P. aeruginosa* and *Haemophilus influenzae*-dominated bacterial communities yielded markedly poorer lung function. More importantly, apart from their higher levels of serum C-reactive protein, sputum interleukin-8 and interleukin-1β, patients whose sputum was predominated by *P. aeruginosa* harbored the lowest levels of lung function and more frequent exacerbations. In our study, we demonstrated that *P. aeruginosa*, but not miscellaneous PPMs, was associated with low FEV_1_ and D_L_CO in unadjusted logistic regression model. The adverse impacts of *Haemophilus influenzae* on lung function were not clearly observed, possibly because of our relatively small sample size. Additionally, McDonnell and associates reported greater persistent infection rates and more hospital admissions associated with *P. aeruginosa*, but not *Haemophilus influenzae* [13]. Apart from high levels of toxins, the heightened airway inflammation leading to mucus plugging might help interpret the mechanisms of dyshomogneity and impaired diffusion. It remained unknown why *P. aeruginosa* isolated from sputum was not related to reduced D_L_CO. A candidate explanation could be that the number of patients with reduced D_L_CO was limited, rendering it less likely to unmask the genuine impacts. However, the adverse impacts of *P. aeruginosa* deserve active antibiotics treatment [14].

Aberrant HRCT features have also been associated with poor lung function. This has been supported by the fact that lung clearance index correlated with chest HRCT scores in patients with bronchiectasis [15]. In the unadjusted regression model, having physician-diagnosed bronchiectasis for 3 years or greater, 24-hour sputum being 30 ml or greater, HRCT total score being 13 or greater, four or more bronchiectatic lobes, dyshomogneity and sputum culture positive for *P. aeruginosa* were concordantly associated with of FEV_1_pred being 50% or less, and D_L_COpred being 80% or less. However, following adjustment of potential confounders, having bronchiectasis symptoms for 10 years or greater, sputum culture positive for *P. aeruginosa* and HRCT total score being 13 or greater, as well as four or more bronchiectatic lobes, were the final variables associated with FEV_1_ 50%pred or less and reduced D_L_CO, respectively. These findings suggested that significantly impaired lung function warrant meticulous history inquiry, chest HRCT and sputum bacteriology. Our findings were partially inconsistent with the report by Ip *et al* [4], which documented that the risk factors consisted of greater sputum volume, older age and concomitant asthma. However, given different study designs and cohorts with different disease severity, it would not be surprising to draw a distinct conclusion. However, it should be acknowledged that our cohort mainly consisted of younger patients with a comparatively low prevalence of asthma, therefore a direct comparison could not be performed.

The changes in spirometry of bronchiectasis exacerbation partially resembled those of chronic obstructive pulmonary disease [16], but not asthma [17], [18]. It was likely that the distinction in pathophysiology that has led to the observed differences in spirometry during exacerbation of these diseases. However, our findings contradicted to the hypothesis that patients with poorer lung function were prone to exhibit more significant reduction in spirometry during bronchiectasis exacerbations. Subgroup analyses by the stratification of the magnitude of FEV_1_ and D_L_CO impairment did not alter our conclusions. Airway inflammation and the resultant mucus hypersecretion were expected to aggravate during exacerbations and recover during convalescence, signaling a dynamic change in airflow obstruction and gas emptying, particularly in patients with poorer lung function when clinically stable. If this presumption was true, the pathophysiology of bronchiectasis exacerbations should be revisited. We next compared small airway indices, which theoretically indicated distal airflow limitation resulting from mucus plugging and airway remodeling. But it was intriguing that significant variations in all subgroups were lacking. The roles of increased mucus secretion or changes in viscosity in the pathogenesis of acute exacerbations remain largely unknown, but it was at least indicated that mucus hypersecretion and airflow limitation had very limited influences on spirometry and that different magnitudes of lung function impairment was not likely to be linked to dramatic changes in bronchiectasis.

Some remarks can be made. A major limitation was the lack of follow-up that determined long-term lung function decline when stratified by the magnitude of FEV_1_ and D_L_CO reduction. Second, since a small proportion of patients were also included in study 2, these results could have reflected the subjects who were less stable and experienced exacerbation during the study, therefore the results might have limited generalisability to the whole spectrum of the disease. Third, the lack of measurement of lung volumes, i.e. residual volume, rendered it impossible to ascertain the degree of air trapping and dyshomogneity. Furthermore, the impacts of acute exacerbations on lung function remained largely unknown. Miscellaneous techniques for measurement of small airway indices (i.e. plethysmography) and multiple-breath nitrogen wash-out technique, may be necessary.

In summary, lung function impairment raises the alert of aberrant chest radiological findings and sputum bacteriology (esp. *P. aeruginosa*) in clinically stable bronchiectasis. Bronchiectasis exacerbations elicit marked reductions in FVC and FEV_1_, but not other spirometric parameters, even when stratified by the magnitude of FEV_1_ and D_L_CO reduction. Further studies regarding the relation between the speed of long-term lung function decline and the magnitude of FEV_1_ and D_L_CO impairment in a larger cohort are warranted.

## Supporting Information

Figure S1 **Changes in spirometric indices in patients colonized with** ***Pseudomonas aeruginosa*** **during steady-state, acute exacerbations and convalescence of bronchiectasis.** Figure S1-A, Changes in FVC; Figure S1-B, Changes in FEV_1_; Figure S1-C, Changes in FEV_1_/FVC; Figure S1-D, Changes in MMEF; Figure S1-E, Changes in MEF_50%_; Figure S1-F, Changes in MEF_25%_.
(TIF)

File S1 **Table S1, Relationship between lung function parameters and clinical indices in clinically stable bronchiectasis.** Data in bold indicated statistical significance. *In our study, bacterial colonization (referred to as infection specifically for P. *aeruginosa*) was defined as sputum culture positive of an identical PPM for at least 2 occasions within 1 year, at least 3 months apart. Bacterial isolation denoted sputum culture positive of PPMs at baseline. **Table S2, Categories of antibiotics for the treatment of acute exacerbations. Table S3, Univariate model for the factors associated with FEV_1_<50%pred and D_L_CO<80%pred.** Data in bold indicated the figures with statistical significance. **Our results showed that the mean logarithm of bacterial load was 7.184, and the geometric median, 7.108. This corresponded approximately to 10−7 cfu/ml, which was therefore elected to be the cut-off for comparison.
(DOC)

Checklist S1 **TREND checklist.**
(DOCX)

Protocol S1 **Study protocol.**
(DOC)
