# Supplementary figures and images for: Characterization of Lung Function Impairment in Adults with Bronchiectasis
Source: PLoS One. 2014 Nov 18;9(11):e113373. doi: 10.1371/journal.pone.0113373 (PMC4236163; doi:10.1371/journal.pone.0113373)

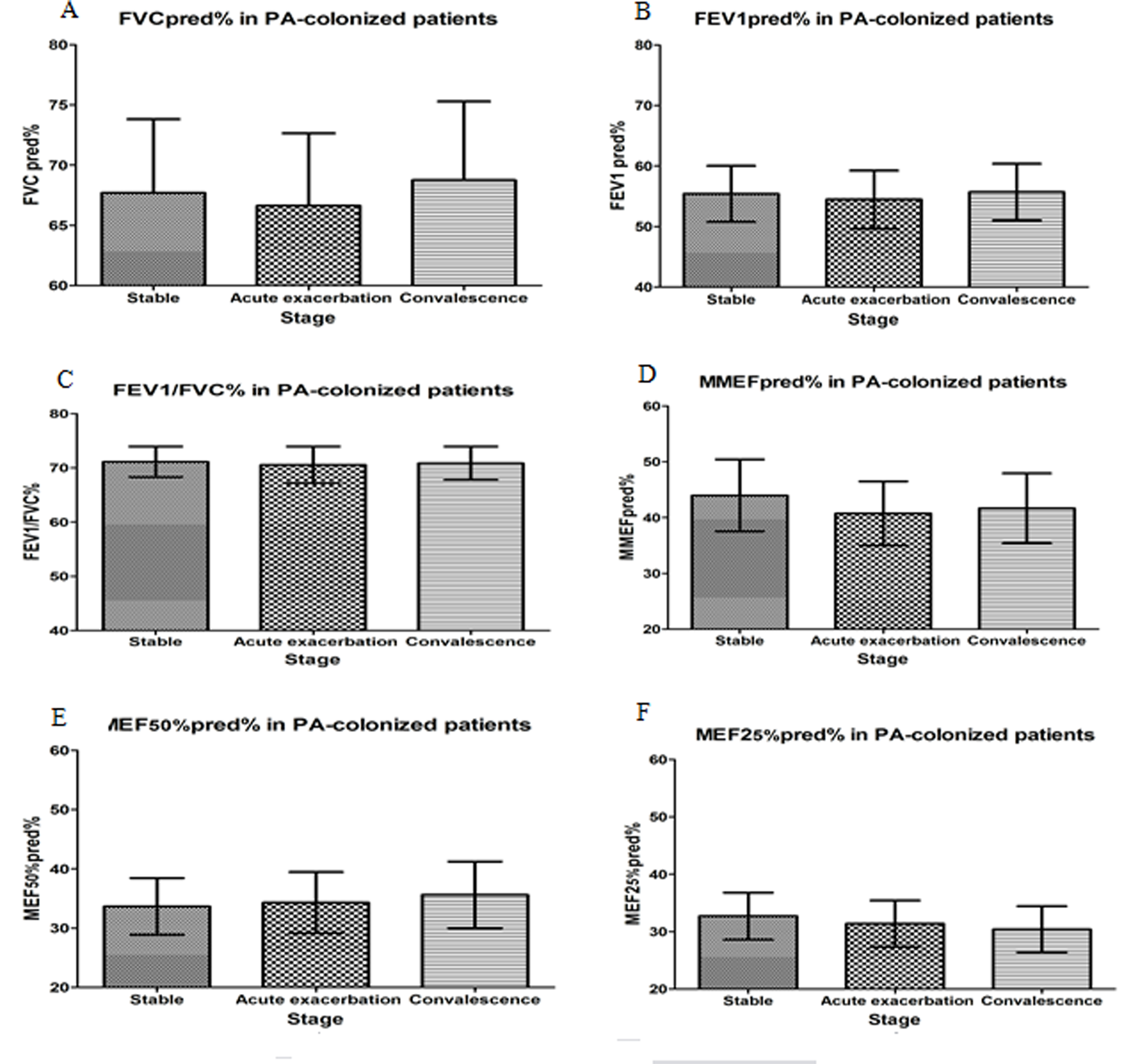

Supplement: Figure S1 — Changes in spirometric indices in patients colonized with Pseudomonas aeruginosa during steady-state, acute exacerbations and convalescence of bronchiectasis. Figure S1-A, Changes in FVC; Figure S1-B, Changes in FEV1; Figure S1-C, Changes in FEV1/FVC; Figure S1-D, Changes in MMEF; Figure S1-E, Changes in MEF50%; Figure S1-F, Changes in MEF25%. (TIF) [file pone.0113373.s001.tif]
