# Supplementary material for: Characterization of Lung Function Impairment in Adults with Bronchiectasis
Source: PLoS One. 2014 Nov 18;9(11):e113373. doi: 10.1371/journal.pone.0113373 (PMC4236163; doi:10.1371/journal.pone.0113373)
Supplement: File S1 — Table S1, Relationship between lung function parameters and clinical indices in clinically stable bronchiectasis. Data in bold indicated statistical significance. *In our study, bacterial colonization (referred to as infection specifically for P. aeruginosa) was defined as sputum culture positive of an identical PPM for at least 2 occasions within 1 year, at least 3 months apart. Bacterial isolation denoted sputum culture positive of PPMs at baseline. Table S2, Categories of antibiotics for the treatment of acute exacerbations. Table S3, Univariate model for the factors associated with FEV1<50%pred and DLCO<80%pred. Data in bold indicated the figures with statistical significance. **Our results showed that the mean logarithm of bacterial load was 7.184, and the geometric median, 7.108. This corresponded approximately to 10−7 cfu/ml, which was therefore elected to be the cut-off for comparison. (DOC) [file pone.0113373.s002.doc]

**Online supplement**

**Characterization of lung function impairment** **in adults with bronchiectasis**

**Wei-jie Guan *1, Ph. D.; Yong-hua Gao *2, Ph. D.; Gang Xu *3, Ph. D.; Zhi-ya Lin 1, Ph. D.; Yan Tang 1, M. D.; Hui-min Li 1, M. T.; Zhi-min Lin 1, M. Med.; Jin-ping Zheng 1, M. D.; Rong-chang Chen 1, M. D.; Nan-shan Zhong 1, M. D.**

1 State Key Laboratory of Respiratory Disease, National Clinical Research Center for Respiratory Disease, First Affiliated Hospital of Guangzhou Medical University, Guangzhou, Guangdong, China

2 Department of Respiratory and Critical Care Medicine, First Affiliated Hospital of Zhengzhou University, Zhengzhou, Henan, China

3 Department of Geriatrics, Guangzhou First People’s Hospital, Guangzhou, Guangdong, China

**Corresponding author 1:** Nan-shan Zhong, M. D., State Key Laboratory of Respiratory Disease, National Clinical Research Center for Respiratory Disease, First Affiliated Hospital of Guangzhou Medical University, Address: 151 Yanjiang Road, Guangzhou, Guangdong, China, Fax: +86-20-83062718, Phone: +86-20-83062718, E-mail: [nanshan@vip.163.com](mailto:nanshan@vip.163.com)

**Corresponding author 2:** Rong-chang Chen, M. D., State Key Laboratory of Respiratory Disease, National Clinical Research Center for Respiratory Disease, First Affiliated Hospital of Guangzhou Medical University, Address: 151 Yanjiang Road, Guangzhou, Guangdong, China, Fax: +86-20-83062719, Phone: +86-20-83062719, E-mail: chenrc99@hotmail.com

*** These authors contributed equally to the study.**

**Short title:** Lung function impairment in bronchiectasis

**Results**

**Relationship between lung function and clinical indices in steady-state bronchiectasis**

Table S1 compared the lung function parameters stratified by clinical indices. When observed with sputum bacteriology, *P. aeruginosa* and *Hemophilus spp* from sputum was associated with poorer FVCpred%, FEV_1_pred% and MMEFpred% (all *P*<0.05). Reduced D_L_COpred%, but not D_L_CO/V_A_pred%, was linked to other PPMs isolated from sputum. Patients with *P. aeruginosa* colonization yielded similar lung function profiles with those with isolation at baseline (both *P*>0.05). *P. aeruginosa* colonization accounted for significantly reduced lung function (*P*<0.05), with exception of FEV_1_/FVC ratio and diffusion (both *P*>0.05).

An increased HRCT score was linked to significantly reduced spirometry and D_L_CO (all *P*<0.05). Four bronchiectatic lobes or more was associated with poorer lung function (all *P*<0.05), except for D_L_CO/V_A_ (*P*>0.05), compared with 3 bronchiectatic lobes or fewer. A similar trend was observed when comparing unilateral with bilateral bronchiectasis, and comparing those who had acute exacerbations of 3 times or fewer with their counterparts. Cystic bronchiectasis, dyshomogneity and pulmonary infiltrations accounted for reduced levels of spirometry (all *P*<0.05), but not diffusing capacity, compared with those without. However, the difference was unremarkable when stratified by upper/lower lobe bronchiectasis and presence of atelectasis (all *P*<0.05).

When observed with other clinical indices, subjects with purulent sputum had worse spirometry (all *P*<0.05), but not diffusing capacity (both *P*>0.05), compared with their counterparts. However, this did not apply for the classification based on 24-hour sputum volume, which demonstrated unmarked differences between all subgroups (*P*>0.05).

**Use of antibiotics for the treatment of acute exacerbation**

The use of antibiotics for the treatment of acute exacerbation is listed in Table S2. The most common type of antibiotic was levofloxacin, followed by amoxicillin/clavulanate potassium. Miscellaneous antibiotics accounted for less than 25% of the total category. All treatment courses were 14 days, as recommended by the British Thoracic Society guidelines for non-cystic fibrosis bronchiectasis.

**Table S1 Relationship between lung function parameters** and clinical indices in clinically stable bronchiectasis

| **Parameter** | **Spirometry (n=142)** | | | | | **Diffusing capacity (n=129)** | | |
| --- | --- | --- | --- | --- | --- | --- | --- | --- |
|  | **No.** | **FVC**  **pred%** | **FEV_1_ pred%** | **FEV_1_/FVC (%)** | **MMEF pred%** | **No.** | **DLCO pred%** | **DLCO/V_A_ pred%** |
| **Sputum bacterial isolation** |  |  |  |  |  |  |  |  |
| *Pseudomonas aeruginosa* | 42 | 69.26±22.08 | 60.49±24.93 | 70.64±13.02 | 45.96±28.11 | 38 | 90.71±13.95 | 108.19 (19.74) |
| *Hemophilus spp* | 27 | 72.05±23.08 | 60.73±24.01 | 70.73±15.04 | 43.93±22.48 | 14 | 95.06±26.03 | 109.00±26.63 |
| Miscellaneous PPMs | 14 | 84.69±6.15 | 76.59±12.83 | 72.95±13.43 | 59.40±29.11 | 14 | 84.85±17.56 | 99.81±18.01 |
| Commensals | 59 | 83.03±18.95 | 77.00±22.21 | 76.03±11.45 | 65.66±32.62 | 53 | 89.05±19.39 | 96.70±14.64 |
| **P value** |  | **0.033** | **0.016** | 0.142 | **0.033** |  | **0.013** | 0.467 |
| **Sputum bacterial colonization*** |  |  |  |  |  |  |  |  |
| *Pseudomonas aeruginosa* | 27 | 68.50±22.13 | 59.50±24.87 | 70.04±11.65 | 44.95±28.13 | 23 | 84.52±17.63 | 107.42±10.58 |
| Nil | 69 | 82.35±19.21 | 75.43±22.49 | 74.98±12.02 | 62.98±32.07 | 61 | 89.85±15.57 | 101.22±14.74 |
| **P value** |  | **0.005** | **0.009** | 0.170 | **0.022** |  | 0.098 | 0.138 |
| **HRCT score** |  |  |  |  |  |  |  |  |
| 1~6 | 70 | 88.26±15.92 | 82.92±18.46 | 77.67±10.37 | 71.78±29.23 | 67 | 92.03 (18.37) | 103.46 (16.40) |
| 7~13 | 57 | 74.14±18.49 | 63.39±21.01 | 70.28±12.67 | 47.21±24.21 | 50 | 89.05±14.52 | 105.17±15.40 |
| 14~18 | 15 | 49.24±14.20 | 37.58±16.92 | 62.56±17.01 | 15.80 (19.76) | 13 | 62.12±20.40 | 95.25±16.81 |
| **P value** |  | **<0.001** | **<0.001** | **<0.001** | **<0001** |  | **<0.001** | 0.160 |
| **HRCT findings** |  |  |  |  |  |  |  |  |
| ≤ 3 bronchiectatic lobes | 66 | 87.91±15.93 | 83.88±18.76 | 78.69±9.91 | 74.04±29.45 | 63 | 96.50±15.92 | 103.58 (17.94) |
| > 4 bronchiectatic lobes | 76 | 70.27±20.84 | 58.69±22.05 | 68.29±13.41 | 42.43±23.83 | 66 | 81.85±18.18 | 102.63±16.32 |
| **P value** |  | **<0.001** | **<0.001** | **<0.001** | **<0.001** |  | **<0.001** | 0.584 |
| Upper and middle lobes predominated | 43 | 80.86±20.95 | 74.31±24.62 | 74.74±13.61 | 62.21±29.86 | 38 | 91.42 (21.66) | 103.52 (15.46) |
| Middle and lower lobes predominated | 99 | 81.53 (20.52) | 68.70±23.77 | 75.70 (12.67) | 54.92±31.17 | 91 | 94.75±14.75 | 102.27±14.12 |
| **P value** |  | 0.365 | 0.204 | 0.329 | 0.197 |  | 0.512 | 0.835 |
| Unilateral bronchiectasis | 26 | 85.86±15.27 | 83.85±16.06 | 81.01±9.37 | 78.44±31.71 | 24 | 93.49±11.49 | 104.95±12.93 |
| Bilateral bronchiectasis | 116 | 81.08 (29.91) | 67.38±24.59 | 71.36±13.03 | 52.35±28.69 | 105 | 91.42 (19.04) | 102.85 (14.73) |
| **P value** |  | **0.043** | **0.001** | **0.001** | **<0.001** |  | 0.190 | 0.601 |
| Presence of cystic bronchiectasis | 78 | 70.79±21.49 | 62.22±25.25 | 75.30 (21.03) | 50.68±32.40 | 69 | 89.78±19.49 | 105.76 (18.62) |
| Nil cystic bronchiectasis | 64 | 87.83±15.06 | 80.37±18.29 | 75.54±10.90 | 64.98±27.09 | 60 | 91.61±15.27 | 100.99±14.49 |
| **P value** |  | **<0.001** | **<0.001** | **0.044** | **0.006** |  | 0.138 | 0.115 |
| dyshomogneity | 91 | 77.77 (32.32) | 62.45±23.40 | 69.91±13.70 | 47.37±27.51 | 80 | 87.49±19.84 | 100.28 (18.69) |
| Nil dyshomogneity | 51 | 84.92±17.85 | 78.56±9.88 | 75.50±28.30 | 62.60±22.89 | 49 | 95.19±14.46 | 104.62±13.53 |
| **P value** |  | **<0.001** | **<0.001** | 0.115 | **0.001** |  | **0.003** | 0.501 |
| Atelectasis | 37 | 73.55±21.31 | 65.07±24.84 | 72.15±14.44 | 50.81±28.42 | 36 | 95.13±14.25 | 103.77±10.09 |
| Nil atelectasis | 105 | 83.38 (26.19) | 72.28±23.64 | 73.47±12.45 | 59.35±31.50 | 93 | 96.42±13.67 | 102.11±14.03 |
| **P value** |  | 0.092 | 0.118 | 0.596 | 0.148 |  | 0.451 | **0.010** |
| Infiltration | 127 | 80.52 (27.68) | 70.04 (33.83) | 72.72±13.39 | 52.28 (45.50) | 114 | 94.02±15.43 | 102.82±13.21 |
| Nil infiltration | 15 | 99.04 (13.85) | 88.56±18.44 | 76.52±8.07 | 74.22±27.66 | 15 | 99.03±14.08 | 106.16±14.76 |
| **P value** |  | **0.001** | **0.002** | 0.285 | **0.023** |  | **0.026** | 0.481 |
| **24-hour sputum volume (ml)** |  |  |  |  |  |  |  |  |
| <10 | 49 | 88.98±13.95 | 80.53±17.49 | 74.68±10.48 | 65.16±27.35 | 45 | 92.19±14.05 | 102.12±14.16 |
| 10~30 | 7 | 67.46±18.08 | 60.33±23.87 | 72.03±17.20 | 50.99±30.13 | 5 | 73.49±24.99 | 99.54±20.56 |
| >30 | 86 | 77.38±21.77 | 65.25±25.80 | 75.80 (19.92) | 53.05±32.19 | 79 | 88.17±20.00 | 104.68 (17.34) |
| **P value** |  | <0.001 | <0.001 | 0.528 | 0.077 |  | 0.082 | 0.659 |
| **Sputum characteristics** |  |  |  |  |  |  |  |  |
| Mucoid | 21 | 86.82±20.50 | 78.94±22.64 | 74.27±11.63 | 61.27±26.71 | 19 | 98.26±21.80 | 102.24 (14.38) |
| Mucopurulent | 24 | 86.86±18.87 | 80.01±24.71 | 80.80 (17.30) | 67.25±32.90 | 22 | 96.79±17.14 | 109.50±14.61 |
| Purulent | 97 | 78.25 (28.39) | 66.00±23.46 | 75.40 (17.80) | 53.72±30.81 | 88 | 85.06±16.70 | 101.46±14.60 |
| **P value** |  | 0.004 | 0.009 | 0.653 | 0.126 |  | 0.002 | 0.096 |
| **No. of exacerbations within 2 yrs** |  |  |  |  |  |  |  |  |
| 0~3 | 76 | 82.02±18.12 | 75.51±21.92 | 78.53±12.60 | 63.90±31.26 | 68 | 93.39±17.51 | 104.47 (18.86) |
| ≥4 | 66 | 74.39±22.66 | 64.48±25.45 | 70.37±13.16 | 49.32±28.68 | 61 | 84.12±18.62 | 101.51±15.53 |
| **P value** |  | 0.028 | 0.006 | 0.018 | 0.005 |  | 0.004 | 0.212 |

Data in bold indicated statistical significance.

* In our study, bacterial colonization (referred to as infection specifically for P. *aeruginosa*) was defined as sputum culture positive of an identical PPM for at least 2 occasions within 1 year, at least 3 months apart. Bacterial isolation denoted sputum culture positive of PPMs at baseline.

**Table S2 Categories of antibiotics for the treatment of acute exacerbations**

| **Category of antibiotics** | **No. (%)** |
| --- | --- |
| Levofloxacin | 28 (63.7) |
| Amoxicillin/clavulanate | 6 (13.6) |
| Cephalosporins | 5 (11.5) |
| Ciprofloxacin | 2 (4.6) |
| Moxifloxacin | 2 (4.6) |
| Piperacillin/tazobactam | 1 (2.3) |

**Table S3 Univariate model for the factors associated with FEV_1_ <50%pred and D_L_CO<80%pred**

| **Parameter** | **FEV_1_ <50%pred** | | | | |  | **D_L_CO <80%pred** | | | | |
| --- | --- | --- | --- | --- | --- | --- | --- | --- | --- | --- | --- |
|  | **Yes (No.)** | **No (No.)** | **Odds ratio** | **95%CI** | **P** |  | **Yes (No.)** | **No (No.)** | **Odds Ratio** | **95%CI** | **P** |
| **Age** |  |  |  |  |  |  |  |  |  |  |  |
| Age≤ 35 | 7 | 31 | 1.00 | - | - |  | 8 | 29 | 1.00 | - | - |
| 35< Age≤ 50 | 9 | 44 | 0.91 | 0.31-2.69 | 0.86 |  | 11 | 37 | 1.08 | 0.38-3.03 | 0.89 |
| Age> 50 | 13 | 38 | 1.52 | 0.54-4.26 | 0.43 |  | 12 | 32 | 1.36 | 0.49-3.79 | 0.56 |
| **Sex** |  |  |  |  |  |  |  |  |  |  |  |
| Male | 11 | 43 | 1.00 | - | - |  | 10 | 39 | 1.00 | - | - |
| Female | 18 | 70 | 1.01 | 0.43-2.33 | 0.99 |  | 21 | 59 | 1.39 | 0.59-3.26 | 0.45 |
| **BMI** |  |  |  |  |  |  |  |  |  |  |  |
| BMI> 24 | 11 | 34 | 1.00 | - | - |  | 14 | 28 | 1.00 | - | - |
| 18.5< BMI≤ 24 | 15 | 62 | 0.75 | 0.31-1.81 | 0.52 |  | 14 | 53 | 0.53 | 0.22-1.26 | 0.15 |
| BMI≤ 18.5 | 3 | 17 | 0.55 | 0.13-2.22 | 0.39 |  | 3 | 17 | 0.35 | 0.09-1.41 | 0.13 |
| **Duration of bronchiectasis symptoms >10 years** |  |  |  |  |  |  |  |  |  |  |  |
| No | 12 | 75 | 1.00 | - | - |  | 16 | 72 | 1.00 | - | - |
| Yes | 17 | 38 | 1.79 | 0.77-4.16 | 0.18 |  | **15** | **26** | **2.60** | **1.13-5.98** | **0.02** |
| **Duration of diagnosis >3 years** |  |  |  |  |  |  |  |  |  |  |  |
| No | 16 | 63 | 1.00 | - | - |  | 15 | 63 | 1.00 | - | - |
| Yes | **13** | **50** | **2.80** | **1.21-6.45** | **0.01** |  | 16 | 35 | 0.52 | 0.23-1.18 | 0.12 |
| **24-h sputum volume (ml)** |  |  |  |  |  |  |  |  |  |  |  |
| <10 | 4 | 45 | 1.00 | - | - |  | 6 | 39 | 1.00 | - | - |
| 11~29 | 2 | 5 | 4.50 | 0.65-31.08 | 0.10 |  | **3** | **2** | **9.75** | **1.34-70.97** | **0.01** |
| >30 | **23** | **63** | **4.11** | **1.33-12.70** | **0.01** |  | 22 | 57 | 2.51 | 0.93-6.75 | 0.06 |
| **No. of exacerbations within 2 yrs** |  |  |  |  |  |  |  |  |  |  |  |
| 0~3 | 12 | 64 | 1.00 | - | - |  | 9 | 59 | 1.00 | - | - |
| ≥4 | 17 | 49 | 1.85 | 0.81-4.23 | 0.14 |  | **22** | **39** | **3.70** | **1.54-8.69** | **0.00** |
| **Sputum characteristics** |  |  |  |  |  |  |  |  |  |  |  |
| Mucoid | 3 | 18 | 1.00 | - | - |  | 1 | 18 | 1.00 | - | - |
| Mucopurulent | 3 | 21 | 0.86 | 0.15-4.79 | 0.86 |  | 3 | 19 | 2.84 | 0.27-29.90 | 0.37 |
| Purulent | 23 | 74 | 1.87 | 0.50-6.90 | 0.35 |  | **27** | **61** | **7.97** | **1.01-62.76** | **0.02** |
| **HRCT total score** |  |  |  |  |  |  |  |  |  |  |  |
| Total score≤ 6 | 5 | 65 | 1.00 | - | - |  | 11 | 56 | 1.00 | - | - |
| 6< total score≤ 12 | **12** | **45** | **3.47** | **1.14-10.52** | **0.02** |  | 12 | 38 | 1.61 | 0.64-4.02 | 0.31 |
| Total score> 12 | **12** | **3** | **52.00** | **10.95-247.02** | **0.00** |  | **8** | **4** | **10.18** | **2.61-39.80** | **0.00** |
| **No. of bronchiectatic lobes** |  |  |  |  |  |  |  |  |  |  |  |
| ≤ 3 | 6 | 61 | 1.00 | - | - |  | 5 | 58 | 1.00 | - | - |
| > 4 | **23** | **52** | **4.50** | **1.70-11.88** | **0.00** |  | **26** | **40** | **7.54** | **2.67-21.30** | **0.00** |
| **Predominantly middle or lower lobe bronchiectasis** |  |  |  |  |  |  |  |  |  |  |  |
| Nil | 8 | 33 | 1.00 | - | - |  | 6 | 30 | 1.00 | - | - |
| Yes | 20 | 78 | 1.06 | 0.42-2.64 | 0.90 |  | 25 | 65 | 1.92 | 0.71-5.18 | 0.19 |
| **Bilateral bronchiectasis** |  |  |  |  |  |  |  |  |  |  |  |
| Nil | 0 | 24 | 1.00 | - | - |  | 2 | 20 | 1.00 | - | - |
| Yes | 28 | 87 | NA | NA | NA |  | 29 | 75 | 3.87 | 0.85-17.60 | 0.06 |
| **Cystic bronchiectasis** |  |  |  |  |  |  |  |  |  |  |  |
| Nil | 3 | 59 | 1.00 | - | - |  | 11 | 47 | 1.00 | - | - |
| Yes | **25** | **52** | **9.46** | **2.70-33.14** | **0.00** |  | 20 | 48 | 1.78 | 0.77-4.12 | 0.18 |
| **dyshomogneity** |  |  |  |  |  |  |  |  |  |  |  |
| Nil | 3 | 45 | 1.00 | - | - |  | 6 | 40 | 1.00 | - | - |
| Yes | **25** | **66** | **4.36** | **1.25-15.23** | **0.01** |  | **25** | **55** | **3.03** | **1.14-8.07** | **0.02** |
| **Atelectasis** |  |  |  |  |  |  |  |  |  |  |  |
| Nil | 17 | 85 | 1.00 | - | - |  | 23 | 67 | 1.00 | - | - |
| Yes | 11 | 26 | 2.12 | 0.88-5.08 | 0.09 |  | 8 | 28 | 0.83 | 0.33-2.08 | 0.70 |
| **Infiltrations** |  |  |  |  |  |  |  |  |  |  |  |
| Nil | 0 | 13 | 1.00 | - | - |  | 1 | 12 | 1.00 | - | - |
| Yes | 28 | 98 | NA | NA | NA |  | 30 | 83 | 4.34 | 0.54-34.80 | 0.14 |
| **Sputum bacteriology** |  |  |  |  |  |  |  |  |  |  |  |
| Commensals | 8 | 51 | 1.00 | - | - |  | 7 | 46 | 1.00 | - | - |
| *Hemophilus spp* | 5 | 22 | 1.45 | 0.43-4.93 | 0.55 |  | 4 | 20 | 1.31 | 0.35-5.00 | 0.69 |
| *Pseudomonas aeruginosa* | **15** | **27** | **3.54** | **1.33-9.41** | **0.01** |  | **16** | **22** | **4.78** | **1.72-13.30** | **0.00** |
| Other PPMs | 1 | 13 | 0.49 | 0.06-4.28 | 0.51 |  | 4 | 10 | 2.63 | 0.64-10.72 | 0.17 |
| **Sputum bacterial load >10^7^cfu/ml *** |  |  |  |  |  |  |  |  |  |  |  |
| No | 17 | 81 | 1.00 | - | - |  | 15 | 57 | 1.00 | - | - |
| Yes | 12 | 32 | 1.02 | 0.45-2.33 | 0.96 |  | 16 | 41 | 1.48 | 0.66-3.34 | 0.34 |
| **Bacterial colonization** |  |  |  |  |  |  |  |  |  |  |  |
| Nil | 16 | 90 | 1.00 | - | - |  | 21 | 76 | 1.00 | - | - |
| Yes | **13** | **23** | **3.18** | **1.34-7.54** | **<0.01** |  | 10 | 22 | 1.65 | 0.68-4.01 | 0.27 |

Data in bold indicated the figures with statistical significance.

** Our results showed that the mean logarithm of bacterial load was 7.184, and the geometric median, 7.108. This corresponded approximately to 10^-7^ cfu/ml, which was therefore elected to be the cut-off for comparison.

**Figure legend**

**Figure S1 Changes in spirometric indices in patients colonized with *Pseudomonas aeruginosa* during steady-state, acute exacerbations and convalescence of bronchiectasis**

Figure S1-A, Changes in FVC; Figure S1-B, Changes in FEV_1_; Figure S1-C, Changes in FEV_1_/FVC; Figure S1-D, Changes in MMEF; Figure S1-E, Changes in MEF_50%_; Figure S1-F, Changes in MEF_25%_.
