# Supplementary material for: Characterization of Lung Function Impairment in Adults with Bronchiectasis
Source: PLoS One. 2014 Nov 18;9(11):e113373. doi: 10.1371/journal.pone.0113373 (PMC4236163; doi:10.1371/journal.pone.0113373)
Supplement: Checklist S1 — TREND checklist. (DOCX) [file pone.0113373.s003.docx]

**TREND checklist**

| **Paper section/topic** | **Item No.** | **Descriptor** | **Reported?** | **Section** |
| --- | --- | --- | --- | --- |
| **Title and abstract** |  |  |  |  |
| Title and abstract | 1 | Information on how units were allocated to interventions | Yes | Title and abstract |
|  |  | Structured abstract recommended | Yes | Abstract |
|  |  | Information on target population or study sample | Yes | Objectives of the Abstract |
| **Introduction** |  |  |  |  |
| Background | 2 | Scientific background and explanation of rationale | Yes | Paragraphs 1 and 2 in introduction section |
|  |  | Theories used in designing behavioral interventions | Yes | Paragraphs 4 in introduction section |
| **Methods** |  |  |  |  |
| Participants | 3 | Eligibility criteria for participants, including criteria at different levels in recruitment/sampling plan (e.g., cities, clinics, subjects) | Yes | Paragraph 1 in Subjects section |
|  |  | Method of recruitment (e.g., referral, self-selection), including the sampling method if a systematic sampling plan was implemented | Yes | Paragraph 1 in Subjects section |
|  |  | Recruitment setting | Yes | Paragraph 1 in Subjects section |
|  |  | Settings and location where the data were collected | Yes | Paragraph 1 in Subjects section |
| Interventions | 4 | Details of the interventions intended for each study condition and how and when they were actually administered, specifically including: |  |  |
|  |  | Content: what was given? | Yes | Paragraphs 1-2 in Study design section |
|  |  | Delivery of method: how was the content given? | Yes | Paragraphs 1-2 in Study design section |
|  |  | Unit of delivery: how were subjects grouped during delivery? |  | Not applicable |
|  |  | Deliverer: who delivered the intervention? | Yes | Paragraphs 1-2 in Study design section |
|  |  | Setting: where was the intervention delivered? | Yes | Paragraphs 1-2 in Study design section |
|  |  | Exposure quantity and duration: how many sessions or episodes or events were intended or to be delivered? How long were they intended to last? | Yes | Paragraphs 1-2 in Study design section |
|  |  | Time span: how long was it intended to take to deliver the intervention to each unit? | Yes | Paragraphs 1-2 in Study design section |
|  |  | Activities to increase compliance or adherence (e.g., incentives) | Yes | Paragraph 2 in Study design section |
| Objectives | 5 | Specific objectives or hypotheses | Yes | Paragraph 5 in Introduction section |
| Outcomes | 6 | Clearly defined primary and secondary outcome measures | No | Not applicable |
|  |  | Methods used to collect data and any methods used to enhance the quality of measurements | Yes | See the Spirometry, Assessment of chest HRCT scores, Measurement of diffusing capacity, 24-hour sputum volume assessment, Sputum culture and quantitative assessment of bacterial load sections |
|  |  | Information on valid instruments such as psychometric and biometric properties | Yes | See the Spirometry, Assessment of chest HRCT scores, Measurement of diffusing capacity, 24-hour sputum volume assessment, Sputum culture and quantitative assessment of bacterial load sections |
| Sample size | 7 | How sample size was determined and, when applicable, explanation of any interim analyses and stopping rules | No | Not applicable |
| Assignment methods | 8 | Unit of assignment (the unit being assigned to study condition, e.g., individual, group, community) | No | Not applicable |
|  |  | Inclusion of aspects employed to help minimize potential bias induced due to non-randomization (e.g., matching) | No | Not applicable |
| Blinding | 9 | Whether or not participants, those administering the interventions, and those were assessing the outcomes were blinded to study condition assignment; if so, statement regarding how the blinding was accomplished and how it was assessed. | No | Not applicable |
| Unit of analysis | 10 | Description of the smallest unit that is being analyzed to assess intervention effects (e.g., individual, group, community) | Yes | See the Spirometry, Assessment of chest HRCT scores, Measurement of diffusing capacity, 24-hour sputum volume assessment, Sputum culture and quantitative assessment of bacterial load sections |
|  |  | If the unit of analysis differs from the unit of assignment, the analytical method used to account for this (e.g., adjusting the standard error estimates by the design effect or using multilevel analysis) | No | Not applicable |
| Statistical methods | 11 | Statistical methods used to compare study groups for primary methods outcome(s), including complex methods or correlated data | Yes | Statistical analysis section |
|  |  | Statistical methods used for additional analysis, such as a subgroup analysis and adjusted analysis | Yes | Statistical analysis section |
|  |  | Methods for imputing missing data, if used | No | Not applicable |
|  |  | Statistical software or program used | Yes | Statistical analysis section |
| **Results** |  |  |  |  |
| Participant flow | 12 | Flow of participants through each stage of the study enrollment, allocation, and intervention exposure, follow-up, analysis (a diagram is strongly recommended) | Yes | Subject recruitment section; Figure 1 |
|  |  | Enrollment: the number of participants screened for eligibility, found to be eligible or ineligible, declined to be enrolled, and enrolled in the study | Yes | Subject recruitment section; Figure 1 |
|  |  | Assignment: the number of participants who completed the follow-up or did not complete the follow-up (i.e., lost to follow-up), by study condition | Yes | Subject recruitment section; Figure 1 |
|  |  | Analysis: the number of participants included or excluded from the main analysis, by study condition | Yes | Subject recruitment section; Figure 1 |
|  |  | Description of protocol deviation from study as planned, along with reasons | No | Not applicable |
| Recruitment | 13 | Dates defining the periods of recruitment and follow-up | Yes | Subjects section of the Introduction |
| Baseline data | 14 | Baseline demographic and clinical characteristics of participant in each study condition | Yes | Paragraph 1 of Baseline levels section |
|  |  | Baseline characteristic of each study condition relevant to specific disease prevention research | Yes | Paragraph 1 of Baseline levels section |
|  |  | Baseline comparisons of those lost to follow-up and those retained, overall and by study condition | No | Not applicable |
|  |  | Comparison between study population at baseline and target population of interest | Yes | Paragraph 1 of Baseline levels section |
| Baseline equivalence | 15 | Data on study group equivalence at baseline and statistical methods used to control for baseline differences | No | Not applicable |
| Numbers analyzed | 16 | Number of participants (denominator) included in each analysis for each study condition, particularly when the denominators change for different outcomes; statement of the results in absolute numbers when feasible | Yes | Tables 2 and 4 |
|  |  | Indication of whether the analysis strategy was “intention to treat” or, if not, description of how non-compliers were treated in the analyses | No | Not applicable |
| Outcomes and estimation | 17 | For each primary and secondary outcome, a summary of results for each estimation study condition, and the estimated effect size and a confidence interval to indicate the precision | Yes | Tables 3 and 4 |
|  |  | Inclusion of null and negative findings | Yes | Table 4 |
|  |  | Inclusion of results from testing pre-specified casual pathways through which the intervention was intended to operate, if any | No | Not applicable |
| Ancillary analyses | 18 | Summary of other analyses performed, including subgroup or restricted analyses, indicating which are pre-specified or exploratory | Yes | See the Association between lung function impairment and clinical indices, Factors associated with FEV1 <50%pred and DLCO<80%pred, and Spirometry during steady-state, acute exacerbation and convalescence of bronchiectasis when stratified by FEV1 and DLCO reduction sections |
| Adverse events | 19 | Summary of all important adverse events or unintended effects in each study condition (including summary measures, effect size estimates, and confidence intervals) | No | Not applicable |
| **Discussion** |  |  |  |  |
| Interpretation | 20 | Interpretation of the results, taking into account study hypotheses, sources of the potential bias, imprecision of measures, multiplicative analyses, and other limitations or weaknesses of the study | Yes | Paragraph 1 in Discussion section |
|  |  | Discussion of results taking into account the mechanism by which the intervention was intended to work (casual pathways) or alternative mechanisms or explanations | Yes | Paragraphs 2, 3 and 4 in Discussion section |
|  |  | Discussion of the success of and barriers to implementing the intervention, fidelity of implementation | Yes | Paragraph 5 in Discussion section |
|  |  | Discussion of research, programmatic, or policy implications | No | Not applicable |
| Generalizability | 21 | Generalizability (external validity) of the trial findings, taking into account the study population, the characteristics of the intervention, length of follow-up, incentives, compliance rates, specific cites/settings involved in the study, and other contextual issues | No | Not applicable |
| Overall evidence | 22 | General interpretation of the results in the context of current evidence and current theory | Yes | Paragraph 6 in Discussion section |
