# Supplementary material for: Characterization of Lung Function Impairment in Adults with Bronchiectasis
Source: PLoS One. 2014 Nov 18;9(11):e113373. doi: 10.1371/journal.pone.0113373 (PMC4236163; doi:10.1371/journal.pone.0113373)
Supplement: Protocol S1 — Study protocol. (DOC) [file pone.0113373.s004.doc]

**Bacteriology and sputum and systemic inflammation in steady-state, acute exacerbation and recovery of bronchiectasis (BISER study)**

**Correspondence 1:** Nan-shan Zhong, M. D., Sate Key Laboratory of Respiratory Disease, First Affiliated Hospital of Guangzhou Medical College. Address: 151 Yanjiang Road, Guangzhou, Guangdong, China, Fax: +86-20-83062718, Phone: +86-20-83062729, E-mail: [nanshan@vip.163.com](mailto:nanshan@vip.163.com)

**Correspondence 2:** Rong-chang Chen, M. D., Sate Key Laboratory of Respiratory Disease, First Affiliated Hospital of Guangzhou Medical College. Address: 151 Yanjiang Road, Guangzhou, Guangdong, China, E-mail: chenrc99@hotmail.com

**Investigators:** Wei-jie Guan, Ph. D. candidate, Zhi-ya Lin, Ph. D., Gang Xu, Ph. D. candidate and Yong-hua Gao, Ph. D. candidate.

1. **Background**

Bronchiectasis is a chronic disease arises from progressive airway inflammation and infection [1]. Pro-inflammatory mediators, the products of activated neutrophils recruited to the inflamed sites, are released in bronchiectatic airways and mediate cascades of neutrophil infiltration [2]. This suggests that bacterial infection plays a pivotal role in the neutrophil-derived inflammation leading to the vicious cycle [3] that perpetuates the development of airway destruction and might result in acute exacerbation. Treatments targeting at bacterial infection is therefore necessary, particularly for those with acute exacerbation of bronchiectasis.

Although short- and long-term administration of antibiotics have been evidenced to markedly suppress bacterial colonization and inflammatory indices [3], the roles that potent antibiotics play in patients with exacerbation of bronchiectasis are unclear. The assessment of bacterial infection and sputum and systemic inflammation during steady-state, acute exacerbation and recovery from exacerbation of bronchiectasis may clinically shed light on and indicate the efficacy of antibiotic treatments.

Furthermore, a subgroup of patients may experience the acute exacerbation that may stem from non-bacterial pathogens. There has been a dire need to compare the changes in sputum bacterial load and inflammatory indices based on sputum bacteriology. This may help uncover the mechanism of different responses to antibiotic treatment in patients who had varying bacteriologic profiles.

Unlike assessment of chronic obstructive pulmonary disease, few clinical indices for appraisal of onset of exacerbation [4] and efficacy [5] of treatments are available. Of these, the 24-hour sputum volume, microbial clearance, C-reactive protein (CRP) and St George’s Respiratory Questionnaire have been validated [5]. In the present study, we employed sputum bacteriology and inflammatory indices, including the aforementioned parameters, for assessment.

1. **Objective**

We aim to determine the changes in bacteriology and sputum and systemic inflammation in steady-state, acute exacerbation and recovery of bronchiectasis. The objectives are four-fold at different time points: (1) sputum microbiology, bacterial load; (2) sputum sol phase inflammatory markers (TNF-α, IL-1, IL-6 and LTB_4_); (3) systemic inflammatory markers (TNF-α, IL-1, IL-6, LTB_4_ and CRP); (4) miscellaneous clinical indices (symptoms, signs and hematology).

1. **Study design**

This is a single-centre, open-label study. Physicians are informed of significant changes in the symptoms or signs (defined as >20% alteration in cough, sputum volume, sputum purulence, wheezing, tachypnea, fever, exercise intolerance and hemoptysis) [6] by telephone in case of potential acute exacerbation. Eligible patients will be treated with a 14-day course of antibiotics based on the sputum culture during steady-state, as recommended by the guideline of British Thoracic Society. Assessment of the recovery phase will be undertaken following accomplishment of antibiotic therapy.

1. **Patient enrollment**

Patients with proven bronchiectasis (as diagnosed by chest high-resolution CT) will be enrolled from First Affiliated Hospital of Guangzhou Medical College.

- 1. The **inclusion criterion** is:

a) Patients of either sex and age between 18 and 70 years.

(2) **Exclusion criteria** encompass:

a) Patient judged to have poor compliance;

b) Female patient who is lactating or pregnant;

c) Patients having concomitant severe systemic illnesses (i.e. coronary heart disease, cerebral stroke, uncontrolled hypertension, active gastric ulcer, malignant tumor, hepatic dysfunction, renal dysfunction);

d) Miscellaneous conditions that would potentially influence efficacy assessment, as judged by the investigators;

e) Participation in another clinical trial within the preceding 3 months.

It is estimated that 80 patients will be recruited in the study. Patients will inform the investigators in the event of an exacerbation when they will be seen within one working day and initiate appropriate antibiotic and other indicated treatment either as an outpatient.

1. **Outcome measures**
2. **Primary outcome measures**

Sputum microbiology (type of bacteria and bacterial load);

1. **Secondary outcome measures**

(1) Serum inflammatory indices (IL-6, TNF-α, LTB_4_, MPO and CRP);

(2) Sputum sol phase inflammatory indices (IL-6, TNF-α, LTB_4_ and MPO)

(3) 24-hour sputum volume;

(4) Spirometric indices (FEV_1_, FVC, FEV_1_/FVC, MMEF);

(5) Sputum purulence (scale 0 to 8);

(6) Sputum viscosity (scale 0 to 3);

(7) SGRQ total score and the score of each domain;

(8) Time to recovery of respective symptom;

(9) Sputum bacterial clearance rate.

1. **Prescription of antibiotics**

Patients will be given antibiotics based on sputum microbiology during steady-state bronchiectasis. The methodology has been described in the British Thoracic Society guideline [7]. Briefly, for first-line therapy, Hemophilus *influenzae*, Hemophilus *parainfluenzae*, Streptoccus *pneumonae* and Moracella *catarrhalis* will be treated with amoxicillin (500mg tid) or amoxicillin clavulanate potassium (625mg bid); Klebsela *pneumonae* or Pseudomonas *aeruginosa* will be treated with fluoroquinolones. Levofloxacin (500mg qd) or moxifloxacin (400mg qd) will be empirically employed for antibiotic treatment in those who test negatively to sputum microbiology. Treatment failure, defined as worsened symptoms or signs or lack of resolve following a 14-day course of oral antibiotic therapy, will lead physicians to initiate intravenous antibiotics therapy, or if warranted, hospitalization for intensive systemic treatment. Subjects requiring hospitalization within or following a 14-day treatment course will be deemed drop-outs in the current study.

1. **Flow chart**

**Steady-state assessments (history inquiry, symptoms and signs, lung function, sputum, blood, etc)**

**Notification by telephone in case of exacerbation**

**Telephone**

**Visit**

**Exacerbation assessments (history inquiry, symptoms and signs, lung function, sputum, blood, etc)**

**Confirmation by symptoms and signs**

**Endpoint reassessments**

**Day 14±1**

**(Visit 3)**

**Baseline**

**Visit 1**

**Dispensing the diary cards**

**Initiation of antibiotics**

**Visit 2**

**Symptoms resolved**

**Treatment ceased**

**Symptoms unresolved**

**Additional treatment**

**Figure 1 Schematic diagram of the study design**

Figure 1 showing a flow chart of subject enrollment and study design. Patients with steady-state bronchiectasis will undergo a baseline assessment, including symptoms, microbiology and inflammatory indices. Investigators will be informed of significant changes in symptoms by telephone in case of potential exacerbation, which will be further confirmed at the hospital visit. Following assessment, patients will be given a 14-course antibiotic therapy, based on BTS guideline. A reassessment of symptoms, sputum microbiology and inflammatory indices will be performed at the end of antibiotic therapy.

1. **Assessments of outcome measures**

**A) Assessment of sputum volume**

Eligible patients with bronchiectasis, following recruitment, will be instructed to record the condition of expectoration in the patient diary card. This includes recording of 24-hour sputum volume, sputum purulence and changes in the symptoms per day. A minimum of 3 daily records between two neighboring visits are required. The 24-hour sputum volume will be recorded as the mean of 3 records. Patients will be constantly followed-up and telephoned, thus securing the compliance, and hence, accuracy and availability of recorded data.

**B) Assessment of inflammatory mediators in sputum sol phase**

Assessment of inflammatory mediators in sputum sol phase has been described previously [8, 9]. Briefly, the fresh sputum sample obtained in the hospital at the visit date was stored in a -70℃ refrigerator and was extracted 15 minutes prior to assessment. This was followed by ultracentrifugation (10000g) at 4℃ for 30 minutes to obtain sputum sol phase. The supernatant was pipetted to a 96-well plate coating with monoclonal antibody of relevant inflammatory mediators. The mixtures were inoculated under room temperature and were abandoned 2 hours later followed by rinsing the plate with buffer solution for three times. Specific enzyme-linked antibodies were then pipetted into the wells and were inoculated for another 2 hours under room temperature followed by thorough rinsing with buffer solution. The substrates were added into the wells and were inoculated for 20 minutes under room temperature for displaying the color. Stop solution (1M sulfuric acid) was added to terminate the reaction. And the concentration of inflammatory mediators was assessed using 450nm spectrometer armed with optical reader. Readings of inflammatory mediators were recorded as pg/ml based on the mean of 3 repetitive measurements.

**C) Assessment of sputum bacterial load**

Assessment of sputum bacterial load has been introduced previously [8, 9].

Briefly, pathogenic bacteria were referred to as Pseudomonas *aeruginosa*, Hemophilus *influenzae*, Streptococcus *pneumonae*, Staphylococcus *aureus*, Moraxella *catarrhalis* and mycobacteria. The culture media for bacterial enrichment included blood agar (Oxoid CM271, containing 5% defibrillated horse serum) and chocolate agar consisted of 18.9mU/ml bacitracin (Sigma Co. Ltd). The fresh sputum specimen was homogenized using SPUTASOL (Oxoid SR089A consisted of 0.1% dithiothreitol, 0.78% sodium chloride, 0.02% potassium chloride, 0.112% disodium hydrophosphate and 0.02% potassium dihydrophosphate) into the serially diluted bacterial mixture (10^1^-, 10^2^- and 10^3^-fold diluted) and was accurately (10 μl) injected onto the culture medium plates by using a pipette. This was followed by inoculation to the corresponding culture media via 10μl standardized inoculation ring. The culture media were positioned in a thermostatic box containing 5% carbon dioxide at 37℃ for overnight inoculation, thus producing a bacterial content of 30-300cfu/ml. All agar plates were divided into four quadrants. Bacterial growth was determined on a daily basis, with a maximum of 4 consecutive days.

**D) Assessment of sputum physical characters [8, 9]**

The 24-hour sputum was assessed in 3 consecutive days. Patients were instructed to expectorate and collect the sputum in a sterile 50ml container followed by storage in a refrigerator at 4℃. Contents in the oral cavity should be removed prior to sputum collection. Patients received chest physical therapy 15 minutes upon arrival at the hospital till expectoration completed. Patients were instructed to be seated and remove contents in the oral cavity followed by sputum collection using a sterile container between 10:00 a.m. and 12:00 a.m., an hour after physical therapy.

The volume of 24-hour sputum was recorded as the mean of the nearest 3 consecutive days. Sputum volume was scored for 1, 2, 3, 4, 5 and 6 points corresponding to 0-10ml, 10-20ml, 20-30ml, 30-40ml, 40-50ml and >50ml, respectively. The central portion of 24-hour sputum was sampled for three aliquots. Sputum purulence was scored for 1, 2, 3, 4, 5, 6 and 7 points corresponding to complete absence, almost translucent, half translucent, translucent but colorless, opaque and white, grey and green, moderately green and dark green, respectively. The specimen with highest score was selected for reports. Sputum viscosity was assessed by using a stick to randomly pick up the sputum from the center of the specimen. Sputum viscosity was scored for 1, 2 and 3 corresponding to mildly, moderately and severely sticky, respectively.

**F)** **Spirometry**

Spirometry tests were carried out using a spirometer (COSMED, QUARK PFT, Italy). All operation procedures met the joint recommendation by ATS and ERS [10]. A total of at least 3 (not more than 8) spirometric maneuvers were performed, with the variation between the best two maneuvers of <5% or 200ml in FVC and FEV_1_. The maximal values of FVC and FEV_1_ were reported. MMEF was chosen from the maneuver with the highest sum of FVC and FEV_1_. The predicted values were selected based on the reference regression model established by Zheng JP and Zhong NS [11].

1. **Patient withdrawal**

Patients might withdraw the study in case of:

- - 1. Unwillingness to continue with the study;
    2. Unexpected deterioration in clinical course either from respiratory or other concurrent illnesses whose management will be facilitated by withdrawal of the antibiotics;
    3. Severe systemic reaction(s) judged by the investigator(s) to be related to administration of the antibiotics.

1. **Use of medication**

**(1) Medications allowed for acute exacerbation**

1. Oral levofloxacin (500mg, q.d., 14 days);
2. Oral moxifloxacin (400mg, q.d., 14 days);
3. Oral amoxicillin (500mg t.i.d., 14 days);
4. Oral amoxicillin clavulanate potassium (625mg b.i.d., 14 days);
5. Oral ciprofloxacin (500mg b.i.d., 14 days).

**(2) Medications abandoned during the study**

1. Intravenous corticosteroids;
2. Miscellaneous inhaled or intravenous antibiotics;
3. Intravenous mucolytics, including ambroxol, bromhexin, serrapeptidase, carbocisteine, 1, 8-cineole, N-acetylcysteine, trypsin and phytpomedicines containing active secretagogue elements;
4. Intravenous xanthene;
5. Nebulized beta-receptor agonist;
6. Nebulized M-receptor antagonist;
7. Miscellaneous medications that might obscure efficacy assessment, as determined by the investigators.

1. **Statistical analysis**

Statistical analysis was performed using SPSS 16.0 version package (SPSS Inc., Chicago, IL.). Test of normality was conducted, and mean ± standard deviation (x±s) was adopted for data with normal distribution, otherwise median (interquartile range) [M(QR)] was used. Two-sided independent t-test was adopted for between-group comparison on end-points when normal distribution was assumed, otherwise non-parametric test was used. Dot plots were applied for comparison on distribution character of inflammatory markers and sputum volume. Row-Column table was analyzed through chi-square test. P<0.05 was taken as statistical significant.

In this study, intention-to-treat (ITT) population included all subjects assigned to antibiotic treatment, regardless of protocol violation. Per-protocol (PP) set is referred to as the patients who sufficiently comply with the protocol to ensure that the data are likely to demonstrate the efficacy of study drug.

1. Expected research progress

| **Time** | **Plans** |
| --- | --- |
| Jun 2012 ~Sep 2012 | Establishment of study protocol |
| Sep 2012~Oct 2013 | Subject enrollment |
| Oct 2013 ~Dec 2013 | Data analysis |
| Dec 2013~Jul 2014 | Manuscript drafting and submission |

1. **Reference**

[1] Baker AF. Bronchiectasis. N Engl J Med. 2002, 346:1383-1393

[2] Fuschillo S, Felice AD, Balzano G. Mucosal inflammation in idiopathic bronchiectasis: cellular and molecular mechanisms. Eur Respir J 2008; 31: 396–406

[3] Chalmers JD, Smith MP, McHugh BJ, et al. Short- and long-term antibiotic treatment reduces airway and systemic inflammation in non-cystic fibrosis bronchiectasis. Am J Respir Crit Care Med. 2012, 1; 186 (7): 657-65

[4] Murray MP, Turnbull K, MacQuarrie S, et al. Assessing response to treatment of exacerbations of bronchiectasis in adults. Eur Respir J. 2009, 33: 312-7

[5] Kapur N, Masters IB and Chang AB. Exacerbations in noncystic fibrosis bronchiectasis: Clinical features and investigations. Respir Med. 2009, 103: 1681-7

[6] Tsang KW, Tan KC, Ho PL, et al. Inhaled fluticasone in bronchiectasis: a 12-month study. Thorax. 2005, 60: 239-53

[7] Pasteur MC, Bilton D, Hill AT, et al. British Thoracic Society guideline for non-CF bronchiectasis. Thorax. 2010, 65: i1-i58

[8] Tsang KW, Ho PL, Lam WK, et al. Inhaled Fluticasone Reduces Sputum Inflammatory Indices in Severe Bronchiectasis. Am J Respir Crit Care Med. 1998;158:723–727

[9] Tsang KW, Chan KN, Ho PL, et al. Sputum Elastase in Steady-State Bronchiectasis. Chest 2000; 117:420–426

[10] Miller MR, Hankinson J, Brusasco V, et al. ATS/ERS task force: Standardization of spirometry. Eur Respir J 2005; 26: 319–338

[11] Zheng JP, Zhong NS. Normative values of pulmonary function testing in Chinese adults. Chin Med J, 2002; 115(1): 50-54

1. **Appendix**

Schedule for follow-up visits

| **Week** | **Steady-state** | **Acute exacerbation** | **Recovery from exacerbation** |
| --- | --- | --- | --- |
| **Day** |  | **0** | **14±1** |
| **Chest high-resolution CT** | **√** |  |  |
| **Complete blood check** | **√** | **√** | **√** |
| **Biochemical screen** | **√** | **√** | **√** |
| **Symptoms** | **√** | **√** | **√** |
| **Signs** | **√** | **√** | **√** |
| **24-hour sputum volume** | **√** | **√** | **√** |
| **Sputum purulence** | **√** | **√** | **√** |
| **Sputum viscosity** | **√** | **√** | **√** |
| **Spirometry** | **√** | **√** | **√** |
| **Sputum bacteriology** | **√** | **√** | **√** |
| **Sputum cytokines** | **√** | **√** | **√** |
| **Serum cytokines** | **√** | **√** | **√** |
| **SGRQ quality of life assessment** | **√** | **√** | **√** |
